# Surgical Treatment Outcomes of Patients with Non-Small Cell Lung Cancer and Lymph Node Metastases †

**DOI:** 10.3390/cancers15123098

**Published:** 2023-06-07

**Authors:** Yuki Shimizu, Terumoto Koike, Toshiki Hasebe, Masaya Nakamura, Tatsuya Goto, Shin-ichi Toyabe, Masanori Tsuchida

**Affiliations:** 1 Division of Thoracic and Cardiovascular Surgery, Niigata University Graduate School of Medical and Dental Sciences, Niigata 951-8510, Japan; 2 Niigata University Crisis Management Office, Niigata University, Niigata 951-8510, Japan

**Keywords:** non-small-cell lung cancer, lymph node metastases, surgical treatment, overall survival, recurrence-free probability, adjuvant chemotherapy

## Abstract

**Simple Summary:**

Multimodal treatment strategies for non-small cell lung cancer (NSCLC) with lymph node metastases are controversial. This study aimed to investigate the risk factors for poor postoperative survival and recurrence in patients with NSCLC and nodal metastasis who were surgically treated and did not receive any induction treatments. Multiple-node metastases are risk factors for both poor survival and recurrence; thus, surgery should be considered for patients with NSCLC and single-node metastasis. Currently, platinum-based adjuvant chemotherapy is considered insufficient in patients with squamous cell carcinoma. Additional perioperative therapies may be required, especially in these patients.

**Abstract:**

This study aimed to investigate the appropriate subgroups for surgery and adjuvant chemotherapy in patients with non-small-cell lung cancer (NSCLC) and nodal metastases. We retrospectively reviewed 210 patients with NSCLC and nodal metastases who underwent surgery and examined the risk factors for poor overall survival (OS) and recurrence-free probability (RFP) using multivariate Cox proportional hazards analysis. Pathological N1 and N2 were observed in 114 (52.4%) and 96 (47.6%) patients, respectively. A single positive node was identified in 102 patients (48.6%), and multiple nodes were identified in 108 (51.4%). Multivariate analysis revealed that vital capacity < 80% (hazard ratio [HR]: 2.678, 95% confidence interval [CI]: 1.483–4.837), radiological usual interstitial pneumonia pattern (HR: 2.321, 95% CI: 1.506–3.576), tumor size > 4.0 cm (HR: 1.534, 95% CI: 1.035–2.133), and multiple-node metastases (HR: 2.283, 95% CI: 1.517–3.955) were significant independent risk factors for poor OS. Tumor size > 4.0 cm (HR: 1.780, 95% CI: 1.237–2.562), lymphatic permeation (HR: 1.525, 95% CI: 1.053–2.207), and multiple lymph node metastases (HR: 2.858, 95% CI: 1.933–4.226) were significant independent risk factors for recurrence. In patients with squamous cell carcinoma (*n* = 93), there were no significant differences in OS or RFP between those who received platinum-based adjuvant chemotherapy (*n* = 25) and those who did not (*n* = 68), at *p* = 0.690 and *p* = 0.292, respectively. Multiple-node metastases were independent predictors of poor OS and recurrence. Patients with NSCLC and single-node metastases should be considered for surgery despite N2 disease. Additional treatment with platinum-based adjuvant chemotherapy may be expected, especially in patients with squamous cell carcinoma.

## 1. Introduction

Lung cancer is among the most commonly diagnosed cancers worldwide and a leading cause of cancer-related deaths [1]. Although surgical resection is a curative treatment for non-small-cell lung cancer (NSCLC), the indications and surgical treatment strategies for locally advanced NSCLC, such as cases with lymph node metastases, are controversial. European and American guidelines [2,3] suggest that multimodal treatment with a multidisciplinary discussion may be considered for locally advanced NSCLCs; however, the details of the recommended multimodal treatment are not clearly mentioned in these guidelines. There are several multimodal treatment options for resectable locally advanced NSCLCs, including preoperative induction chemotherapy with or without radiation therapy, postoperative adjuvant chemotherapy, and postoperative radiation therapy. In addition, multimodal treatment has recently become more complex, with more options, such as adjuvant tyrosine kinase inhibitors [4] and neoadjuvant or adjuvant immune checkpoint inhibitors [5,6]. Since patients who underwent pneumonectomy after induction chemoradiotherapy did not show a survival benefit compared to those who underwent definitive chemoradiotherapy without surgery [7], the extent of pulmonary resection required for complete resection may affect the assessment of resectability and indications for surgical resection for such NSCLCs.

In the current study, we retrospectively reviewed the data of patients with NSCLC and histologically proven lymph node metastases who underwent surgical resection without any treatment prior to surgery. This study aimed to investigate the appropriate subgroups for surgical resection in patients with NSCLC and lymph node metastases by identifying the independent demographic, clinical, and pathological factors associated with postoperative survival and recurrence. Moreover, we examined the effects of postoperative adjuvant chemotherapy on outcomes in these patients.

## 2. Materials and Methods

**Figure 1:**
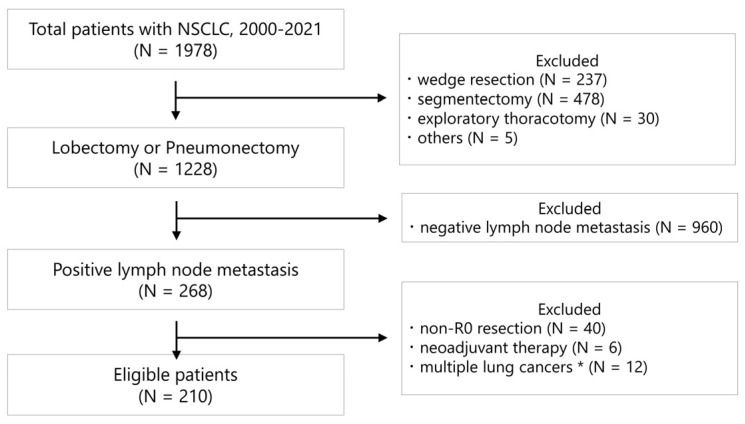
CONSORT-style diagram illustrating the selection of patients included in the data analysis. * Patients with synchronous lung cancer and a history of lung cancer treatment.

A total of 1978 patients with NSCLC underwent surgery between January 2000 and December 2021 at Niigata University Medical and Dental Hospital. We retrospectively reviewed 210 consecutive patients who underwent lobectomy or pneumonectomy and had pathological lymph node metastases. Patients who underwent non-R0 resection and neoadjuvant therapy, had multiple lung cancers defined as synchronous cancers, or had a previous treatment history for lung cancer were excluded from the analysis (Figure 1).

All patients underwent contrast-enhanced chest and abdominopelvic computed tomography (CT) and enhanced magnetic resonance imaging (MRI) or brain CTs for preoperative staging. The 18-fluorodeoxyglucose positron emission tomography/computed tomography (FDG-PET/CT) modality was introduced to our institution in July 2015, and all patients have undergone this test since its introduction. Bone scintigraphy was performed prior to the introduction of FDG-PET/CT. Invasive modalities for mediastinal lymph node staging were used at the discretion of the attending surgeon or physician to determine the treatment strategy.

The standard surgical procedure at our institution is lobectomy with mediastinal lymph node dissection, and bilobectomy or pneumonectomy is selected if necessary for complete resection. Segmentectomy was selected for patients with cN0 and tumors ≤ 2 cm [8]. Lymph node stations were recorded according to the definitions of the International Association for the Study of Lung Cancer [9]. Based on previous studies demonstrating improved postoperative outcomes in patients with completely resected node-positive NSCLC treated with platinum-based adjuvant chemotherapy [10], postoperative platinum-based chemotherapy was offered to these patients. Postoperative platinum-based adjuvant chemotherapy has been offered in our institution since 2005 [10]. If postoperative platinum-based adjuvant chemotherapy was inappropriate for various reasons, such as age ≥ 75 years, renal dysfunction, comorbid diseases, or patient refusal, oral tegafur-uracil (UFT) was considered [11]. Cytotoxic chemotherapy, such as platinum-containing drugs for patients with lung cancer with comorbid interstitial pneumonia, has a high risk of acute exacerbation [12], especially with the radiological usual interstitial pneumonia (UIP) pattern [13]. Therefore, postoperative platinum-based adjuvant chemotherapy was not indicated in patients with radiological interstitial pneumonia.

All patients who underwent surgery were followed up in the outpatient department with blood chemistry tests, including tumor markers, and imaging studies, including chest radiography and chest CT. Chest CT was performed at least yearly. Additional imaging, such as brain MRI and FDG-PET/CT, was performed at the discretion of the attending surgeon if recurrence was suspected based on routine radiological and laboratory examinations. Recurrence was determined based on imaging, and a pathological diagnosis of recurrent lesions was not mandatory.

Clinical factors analyzed were age (≤69 years or >69 years), sex, smoking status (Brinkman index [14]; ≤600 or >600), percentage predicted vital capacity (%VC) (<80% or ≥80%), forced expiratory volume in one second/forced expiratory volume (Fev1.0%) (<70% or ≥70%), radiological UIP pattern (UIP and possible UIP pattern, or others [15]), preoperative serum carcinoembryonic antigen (CEA) level (≤5 ng/mL or >5 ng/mL), diseased side (right or left), tumor location (upper, middle or lower lobe), surgical procedure (lobectomy or pneumonectomy), the extent of mediastinal lymph node dissection (selective or systematic [16]), and adjuvant chemotherapy (platinum-based chemotherapy or others). Pathological factors analyzed were tumor size (≤4.0 cm or >4.0 cm [17]), histology (adenocarcinoma, squamous cell carcinoma, or others), lymph node metastasis status (pN1 or pN2 [17]), visceral pleural invasion (absent or present), lymphatic permeation (absent or present), vascular invasion (absent or present), number of positive lymph nodes (single or multiple), number of positive lymph node stations (single station or multiple stations), positive lymph node ratio (number of positive lymph nodes/number of resected lymph nodes [≤0.09 or >0.09]) and epidermal growth factor receptor (EGFR) mutation status (positive, negative, or unknown). Regarding age and positive lymph node ratio, we divided the patients based on the median. The cutoff for the normal upper limit of CEA was set at 5 ng/mL. Regarding smoking status, a Brinkman index of over 600 was defined as a heavy smoker [14]. The cutoffs at the normal lower limits of %VC and Fev1.0% were 80% and 70%, respectively [18]. Visceral pleural invasion, lymphatic permeation, and vascular invasion were evaluated by pathologists using hematoxylin, eosin, and Elastica van Gieson staining.

The length of overall survival was estimated as the interval between the date of surgery and the date of any cause of death or last follow-up. Recurrence-free probability was measured from the date of surgery to the date of the first recurrence or last follow-up. For univariate analyses, overall survival and recurrence-free probability were calculated using the Kaplan–Meier method, and the risk factors for poor postoperative survival and recurrence were analyzed using the log-rank test. In multivariate analyses, all factors were examined using the Cox proportional hazards model. A stepwise method was used to determine the independent risk factors for poor postoperative survival and recurrence. Statistical significance was set at *p* < 0.05. All analyses were performed using IBM SPSS 26 software (IBM Co., Chicago, IL, USA).

## 3. Results

Of the 210 patients included in this study, 160 were male, and 50 were female. The median age was 69 years (interquartile range [IQR]: 63–74 years). The median tumor size was 3.8 cm (IQR: 2.5–5.0 cm). The tumor histology was adenocarcinoma in 91 patients (43.3%), squamous cell carcinoma in 93 (44.3%), and others in 26 (12.4%). The other histological types included pleomorphic carcinoma in six patients, adenosquamous carcinoma in six, large cell carcinoma in five, large cell neuroendocrine carcinoma in four, carcinoid tumor in two, salivary gland-type tumor in two, and unclassified carcinoma in one. Lobectomies, including bilobectomies, were performed in 200 patients (95.2%) and pneumonectomies were performed in 10 patients (4.8%). According to the current TNM classification [17], pathological N1 and N2 were observed in 114 (52.4%) and 96 (47.6%) patients, respectively. Of the 114 patients with pathological N1, clinical N0, N1, and N2 were observed in 53, 57, and 4 patients, respectively. Of the 96 patients with pathological N2, clinical N0, N1, and N2 were observed in 39, 29, and 28 patients, respectively. A single positive lymph node was observed in 102 patients (48.6%) and multiple positive lymph nodes were observed in 108 patients (51.4%). Single positive lymph node stations were identified in 115 patients (54.8%) and multiple stations were observed in 95 patients (45.2%). The median numbers of positive lymph nodes, positive lymph node stations, and resected lymph nodes were 2 (IQR: 1–3), 1 (IQR: 1–2) and 19 (IQR: 15–27), respectively. The median positive lymph node ratio was 0.09 (IQR: 0.06–0.18). Of all patients, 75 (35.7%) received platinum-based adjuvant chemotherapy, 60 received cisplatin-based chemotherapy and 15 received carboplatin-based chemotherapy. UFT was given to 5 (2.4%) patients, and 2 (1.0%) received postoperative radiotherapy. Furthermore, 128 (60.9%) patients did not receive any postoperative adjuvant therapy, including 28 patients who underwent surgery before 2005, 32 patients with radiological interstitial pneumonia, and 42 patients > 75 years old. Other reasons for not receiving adjuvant therapy were renal dysfunction, comorbid diseases, and patient refusal.

**Figure 2:**
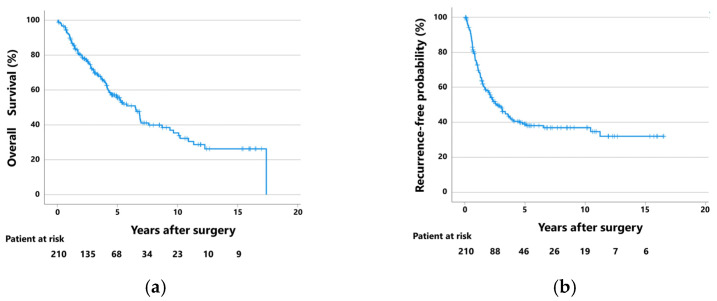
(**a**) Overall survival curves and (**b**) recurrence-free probability curves of the 210 patients with pathological lymph node metastases who were surgically treated with lobectomy or pneumonectomy.

The 5-year overall survival (OS) rate was 55.6% at a median follow-up of 44 months (IQR: 21–68 months), and the 5-year recurrence-free probability (RFP) was 39.0% at a median follow-up of 24 months (IQR: 10–55 months) (Figure 2). During the follow-up period of 44 months, 117 patients (55.7%) developed recurrence; 31 of these had locoregional recurrence, 36 had distant recurrences, and 50 had both. Treatment for recurrence was given to 100 patients (85.5%) with postoperative recurrence, and 17 did not receive any treatment because of impaired general conditions and comorbidities.

Univariate analysis identified %VC, radiological UIP pattern, adjuvant chemotherapy, tumor size, histology, lymphatic permeation, number of lymph node metastases, number of positive lymph node stations, and positive lymph node ratio as factors associated with OS (Table 1). The multivariate analysis revealed that %VC < 80% (hazard ratio [HR]: 2.678, 95% confidence interval [CI]: 1.483–4.837), radiological UIP pattern (HR: 2.321, 95% CI: 1.506–3.576), tumor size > 4.0 cm (HR: 1.534, 95% CI: 1.035–2.133), and multiple lymph node metastases (HR: 2.283, 95% CI: 1.517–3.955) were significant independent risk factors for poor OS (Table 2). Univariate analysis of RFP showed that preoperative CEA levels, tumor size, lymph node metastasis status, lymphatic permeation, number of positive lymph nodes, number of positive lymph node stations, and positive lymph node ratio were associated with postoperative recurrence (Table 1). In the multivariate analysis for RFP, tumor size > 4.0 cm (HR: 1.780, 95% CI: 1.237–2.562), lymphatic permeation (HR: 1.525, 95% CI: 1.053–2.207), and multiple lymph node metastases (HR: 2.858, 95% CI: 1.933–4.226) were identified as significant independent risk factors for postoperative recurrence (Table 3).

**Figure 3:**
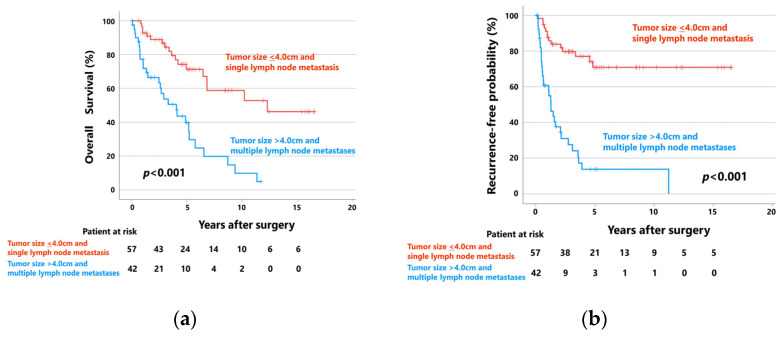
(**a**) Overall survival curves and (**b**) recurrence-free probability curves of patients with tumors ≤ 4.0 cm and single lymph node metastases (*n* = 57), and those with tumors > 4.0 cm and multiple lymph node metastases (*n* = 42).

Patients with tumors ≤ 4.0 cm and single lymph node metastases (*n* = 57) showed significantly better OS and RFP than those with tumors > 4.0 cm and multiple lymph node metastases (*n* = 42) (Figure 3). The 5-year OS rates of patients with tumors ≤ 4.0 cm and single lymph node metastases and those with tumors > 4.0 cm and multiple lymph node metastases were 71.4% (median follow-up: 55 months) and 39.7% (median follow-up: 28 months), respectively (*p* < 0.001). The 5-year RFPs of these groups were 70.9% (median follow-up: 46 months) and 13.8% (median follow-up: 11 months), respectively (*p* < 0.001).

**Figure 4:**
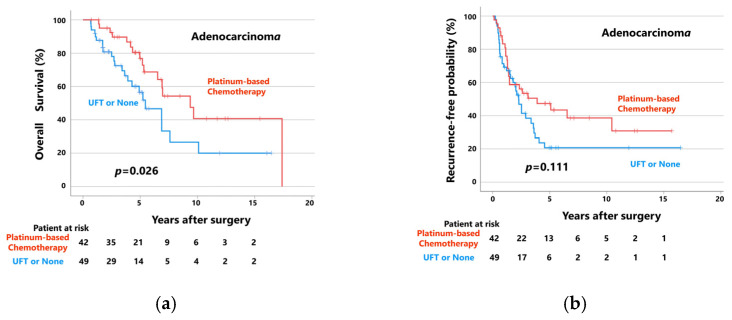

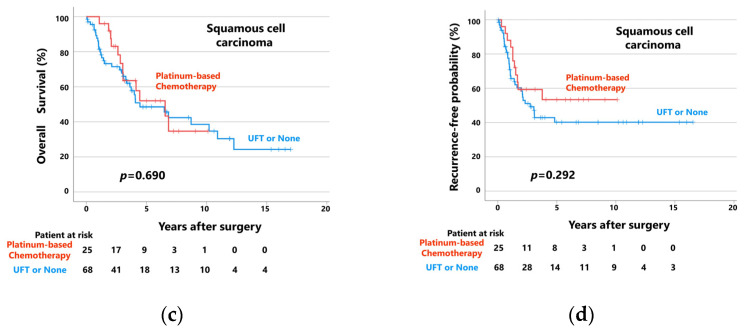
(**a**) Overall survival curves and (**b**) recurrence-free probability curves of patients with adenocarcinoma, patients who received platinum-based chemotherapy, and patients who received UFT or did not receive adjuvant chemotherapy. (**c**) Overall survival curves and (**d**) recurrence-free probability curves of patients with squamous cell carcinoma, patients who received platinum-based chemotherapy, and patients who received UFT or did not receive adjuvant chemotherapy.

Figure 4 shows the OS (a) and RFP (b) curves of patients with adenocarcinoma (*n* = 91) who received postoperative platinum-based adjuvant chemotherapy (*n* = 42) and those who received UFT or did not receive adjuvant chemotherapy (*n* = 49). Patients who received postoperative platinum-based adjuvant chemotherapy (median follow-up: 61 months) showed significantly better OS than those who did not (median follow-up: 40 months). The 5-year OS rates of the patients with adenocarcinoma who did and did not receive postoperative platinum-based adjuvant chemotherapy were 76.8% and 56.6%, respectively (*p* = 0.026). Moreover, patients who received postoperative platinum-based adjuvant chemotherapy (median follow-up: 31 months) tended to have better RFPs than those who did not (median follow-up: 22 months). The 5-year RFPs of these groups were 47.4% and 20.7%, respectively (*p* = 0.111).

However, when patients with squamous cell carcinoma (*n* = 93) were divided into two groups according to postoperative adjuvant chemotherapy status, there were no significant differences in OS or RFP between patients who received postoperative platinum-based adjuvant chemotherapy (*n* = 25) and those who did not (*n* = 68; Figure 4c,d). In patients who did and did not receive platinum-based adjuvant chemotherapy, the 5-year OS rates and RFPs were 52.0% and 48.5%, respectively (median follow-up: 39 and 39 months, respectively; *p* = 0.690) and 53.4% versus 40.2%, respectively (median follow-up: 25 and 19 months, respectively; *p* = 0.292).

## 4. Discussion

In this study, we retrospectively analyzed the independent risk factors for poor OS and postoperative recurrence in 210 patients with node-positive lung NSCLC who underwent surgery. Multiple lymph node metastases and tumor size > 4.0 cm were identified as independent risk factors for poor OS and postoperative recurrence. Moreover, %VC < 80% and a radiological UIP pattern were identified as independent prognostic factors for poor OS, and lymphatic permeation was identified as a risk factor for postoperative recurrence.

Patients with adenocarcinoma who received postoperative platinum-based adjuvant chemotherapy showed significantly better OS and tended to show better RFP than those who did not. In contrast, in patients with squamous cell carcinoma, there were no significant differences in either OS or RFP between the patients who received postoperative platinum-based adjuvant chemotherapy and those who did not.

Although the N factor has a significant impact on staging and prognosis in the TNM classification [17], the N descriptor is determined only by the anatomic extent of the involved lymph nodes. As lymph node metastases theoretically spread along the lymphatic tracts, there is a possibility that the number of metastatic lymph nodes is also related to postoperative outcomes. Many studies have suggested that the number of lymph node metastases may have a prognostic potential in NSCLC. Lee et al. reported that when patients were divided into four groups according to the number of metastatic lymph nodes, with nodal metastases of 0, 1–3, 4–14, and ≥15, this classification showed a stratified prognosis and was associated with OS [19]. Other studies have reported that the number of metastatic lymph nodes is an independent prognostic factor in patients with NSCLC and hilar [20] and mediastinal node metastases [21].

Because the number of metastatic lymph nodes is affected by the number of resected lymph nodes, Kai et al. investigated the effect of a positive lymph node ratio on postoperative outcomes [22]. They divided patients into three groups according to the lymph node ratio (≤0.28, 0.28–0.81, and ≥0.81) and showed that a positive lymph node ratio was an independent and significant adverse predictor of cancer-specific death in NSCLC. Katsumata et al. analyzed the prognosis of patients with NSCLC using a combination of the current 8th-edition N classification [17] and the number of positive lymph nodes [23]. In their report, multiple N1 metastases showed a significantly worse prognosis than a single N1 metastasis, and there were no significant differences in OS among patients with multiple N1 metastases, single N2 metastasis, or multiple N2 metastases.

Similar results were reported by Wei et al. and Saji et al. [24,25]. These studies indicated that the number of metastatic lymph nodes had a greater impact on prognosis than the N1 and N2 classifications. The present study showed that the number of positive lymph nodes might be a better independent factor for poor postoperative survival and recurrence in patients with NSCLC and lymph node metastases than the current N classification in the TNM staging system [17]. In addition, our results showed that the number of positive lymph nodes had a more significant impact than the number of positive lymph node stations and the positive lymph node ratio on the association between poor postoperative outcome and recurrence.

Thus, we believe that patients with NSCLC and single-node metastases should be considered for surgery, even if they have N2 disease. Recently, improved postoperative outcomes have been suggested by using novel postoperative adjuvant systemic treatment strategies using osimertinib and atezolizumab [4,6]. In these studies, the 2-year disease-free survival of the patients with stage II to IIIA EGFR-mutated NSCLC in the osimertinib group was 90% [4], and the 2-year disease-free survival of the patients with stage II to IIIA NSCLC in the atezolizumab group was 71.4% [6]. In our study, the 2-year RFP of the patients with tumors ≤ 4.0 cm and single lymph node metastases was 83.9%. Although an indirect comparison, this result does not seem significantly inferior to that of patients who underwent novel adjuvant systemic treatments using immune checkpoint inhibitors and tyrosine kinase inhibitors. The low-risk subgroups of patients identified in this study may be potential candidates when seeking a subgroup of patients who do not require postoperative adjuvant chemotherapies.

Surgical resection is the only curative treatment for early-stage NSCLC; however, it is reported that 30–55% of patients develop recurrence after surgical resection [26]. The reason for recurrence despite complete resection is assumed to be the presence of micrometastases that could not be detected in preoperative imaging at the time of surgery. It has also been reported that surgical manipulation may spread tumor cells into the bloodstream [27]. This is based on the hypothesis that adjuvant chemotherapy for micrometastases after complete resection enhances the therapeutic effect. Three randomized phase 3 trials, IALT [10], JBR.10 [28], and ANITA [29], and a meta-analysis of data from 4584 patients, the LACE meta-analysis [30], showed improvements in disease-free survival and OS with cisplatin-based adjuvant chemotherapy after surgical resection for locally advanced NSCLC, such as lymph node metastases. These strategies for postoperative adjuvant chemotherapies have a survival benefit in patients with stage II and stage IIIA NSCLC; thus, platinum-based adjuvant chemotherapy is recommended for patients with resected stage II and III NSCLC according to current guidelines [2,3,31].

In our study, adjuvant chemotherapy seemed to improve OS and RFP in patients with adenocarcinoma but not in patients with squamous cell carcinoma. Poor efficacy of adjuvant chemotherapy in patients with squamous cell carcinoma has been reported [32], and a subgroup analysis of the LACE meta-analysis showed less prognostic improvement with platinum-based adjuvant chemotherapy in patients with squamous cell carcinoma than in those with adenocarcinoma [30].

In recent years, immune checkpoint inhibitors have provided a breakthrough in cancer treatment. The Impower010 trial demonstrated that atezolizumab after platinum-based adjuvant chemotherapy improved disease-free survival among patients with stage II to IIIA resected NSCLC, especially in the PD-L1 ≥ 1% [6]. The CheckMate 816 trial demonstrated that patients with resectable NSCLC who received neoadjuvant nivolumab plus chemotherapy had significantly longer event-free survival than those who received chemotherapy only [5]. A previous study indicated that PD-L1 expression was more frequent in patients with squamous cell carcinoma than in those with adenocarcinoma [33]. Our results suggested that in patients with squamous cell carcinoma, platinum-based adjuvant chemotherapy might be insufficient when compared to those with adenocarcinoma. Thus, novel multimodality treatment strategies for locally advanced NSCLC, such as adjuvant or neoadjuvant immune checkpoint inhibitors, may contribute to the survival benefit considered in patients with squamous cell carcinoma.

This study had some limitations. This was a retrospective study with a small sample size and was conducted at a single institution. There were some biases due to the patients’ backgrounds, especially regarding whether adjuvant chemotherapy was administered. In addition, because of the broad coverage period, the modalities for preoperative assessment were inconsistent. Although preoperative evaluation using FDG-PET/CT and pathological assessment for suspected nodal metastasis are strongly recommended in patients with advanced-stage NSCLC in the current guidelines [2,3], FDG-PET/CT was not used before July 2015, and endoscopic bronchial ultrasound and mediastinoscopy were often omitted at the discretion of the attending surgeon or physician. Another limitation was that recurrence was determined primarily based on imaging and mostly not confirmed in histology. However, we believe that our results will be helpful in deciding the indications for surgery and perioperative chemotherapy by identifying the risk factors for recurrence and poor survival in patients with NSCLC and lymph node metastasis.

## 5. Conclusions

We retrospectively reviewed 210 patients with NSCLC and lymph node metastases who were surgically treated with lobectomy or pneumonectomy and examined the risk factors for poor postoperative survival and recurrence using univariate and multivariate analyses. Multiple lymph node metastases were an independent predictor of both poor postoperative survival and recurrence. Thus, we believe that patients with NSCLC and single-node metastases should be considered for surgical treatment even if they have N2 disease. Platinum-based adjuvant chemotherapy seemed to improve postoperative outcomes in patients with adenocarcinoma, but not in patients with squamous cell carcinoma. Novel multimodal treatment strategies for patients with NSCLC and lymph node metastases, such as neoadjuvant and adjuvant systemic treatments using immune checkpoint inhibitors, may be useful, especially in patients with squamous cell carcinoma.

## Figures and Tables

**Table 1:** Baseline characteristics and univariate analysis of overall survival and recurrence-free probability for patients with pathological lymph node metastases who were surgically treated with lobectomy or pneumonectomy.

Variable	No. of Patients (%)	5-Year Overall Survival (%)	*p*-Value	5-Year Recurrence-Free Probability (%)	*p*-Value
All cases	210 (100)	55.6		39.0	
Age					
≤69 years	114 (54)	54.7	0.696	40.8	0.884
>69 years	96 (46)	57.6		36.8	
Sex					
Male	160 (76)	49.9	0.057	41.5	0.304
Female	50 (24)	74.8		31.7	
Smoking status (BI)					
≤600	65 (30)	68.6	0.101	29.4	0.280
>600	145 (70)	49.6		44.3	
%VC					
<80%	20 (10)	15.2	**<0.001**	38.8	0.337
≥80%	190 (80)	59.3		39.4	
Fev1.0%					
<70%	60 (29)	57.5	0.443	32.8	0.196
≥70%	150 (71)	50.8		41.6	
Radiological UIP pattern					
UIP, possible UIP	43 (20)	32.9	**<0.001**	41.4	0.990
Others	167 (80)	61.4		38.3	
CEA					
≤5 ng/mL	91 (43)	65.7	0.067	46.2	**0.034**
>5 ng/mL	119 (57)	48.0		33.4	
Diseased side					
Right	127 (60)	54.7	0.566	39.8	0.524
Left	83 (40)	56.4		37.9	
Tumor location					
Upper/middle lobe	116 (55)	61.1	0.096	43.0	0.153
Lower lobe	94 (45)	48.6		33.9	
Surgical procedure					
Lobectomy	200 (95)	56.7	0.566	39.7	0.693
Pneumonectomy	10 (5)	36.0		30.0	
Mediastinal lymph node dissection					
selective	58 (28)	56.7	0.923	42.0	0.482
systematic	152 (72)	55.3		38.0	
Adjuvant Chemotherapy					
Platinum-based Chemotherapy	75 (36)	67.7	**0.020**	49.9	0.143
others	135 (64)	48.4		32.3	
Pathological tumor size					
≤4.0 cm	123 (59)	59.8	**0.035**	46.2	**0.006**
>4.0 cm	87 (41)	49.5		28.4	
Tumor histology					
Adenocarcinoma	91 (43)	66.3	**0.036**	33.9	0.363
Squamous cell carcinoma	93 (44)	49.7		43.6	
Others	26 (13)	39.6		41.9	
pN status					
N1	114 (54)	58.8	0.365	47.6	**0.004**
N2	96 (46)	51.8		29.5	
Pleural invasion					
Absent	100 (48)	54.6	0.864	38.5	0.310
Present	110 (52)	56.6		39.1	
Lymphatic permeation					
Absent	135 (64)	61.2	**0.048**	43.4	**0.004**
Present	75 (36)	45.1		31.1	
Vascular invasion					
Absent	129 (61)	57.9	0.159	39.6	0.132
Present	81 (39)	52.3		37.6	
No. of positive LNs					
single	102 (49)	65.3	**<0.001**	57.7	**<0.001**
multiple	108 (51)	46.2		21.0	
No. of positive LN stations					
single	115 (55)	63.7	**0.003**	53.6	**<0.001**
multiple	95 (45)	46.5		21.9	
positive LN ratio					
≤0.09	106 (50)	63.6	**0.005**	53.0	**<0.001**
>0.09	104 (50)	46.9		24.1	
EGFR mutation					
negative	123 (59)	50.9	0.497	42.3	0.076
positive	39 (19)	71.8		20.9	
unknown	48 (22)	54.8		46.7	

BI: Brinkman Index: calculated as the number of cigarettes smoked/day multiplied by the number of years since smoking started. CEA: preoperative serum carcinoembryonic antigen level. %VC: predicted Vital Capacity. Fev1.0%: Forced expiratory volume in one second/forced expiratory volume. UIP: usual interstitial pneumonia. LN: lymph node. Positive LN ratio: positive lymph node ratio: the number of positive lymph nodes/the number of resected lymph nodes. EGFR: epidermal growth factor receptor. *p*-values in bold are statistically significant.

**Table 2:** Multivariate Cox proportional hazard analysis of overall survival in patients with pathological lymph node metastases who were surgically treated with lobectomy or pneumonectomy.

Variable	Hazard Ratio	95% CI	*p*-Value
%VC			
<80%	2.678	1.483–4.837	0.001
≥80%	Reference		
Radiological UIP pattern			
UIP, possible UIP	2.321	1.506–3.576	<0.001
Others	Reference		
Pathological tumor size			
≤4.0 cm	Reference		
>4.0 cm	1.534	1.035–2.133	0.033
No. of positive LNs			
single	Reference		
multiple	2.283	1.517–3.955	<0.001

CI: confidence interval. %VC: predicted Vital Capacity. UIP: usual interstitial pneumonia. LN: lymph node.

**Table 3:** Multivariate Cox proportional hazard analysis of recurrence-free probability in patients with pathological lymph node metastases who were surgically treated with lobectomy or pneumonectomy.

Variable	Hazard Ratio	95% CI	*p*-Value
Pathological tumor size			
≤4.0 cm	Reference		
>4.0 cm	1.780	1.237–2.562	0.002
Lymphatic permeation			
Absent	Reference		
Present	1.525	1.053–2.207	0.025
No. of positive LNs			
single	Reference		
multiple	2.858	1.933–4.226	<0.001

CI: confidence interval. LN: lymph node.

## Data Availability

Data is available on reasonable request from the authors.
